# Evaluation of non-linear heart rate variability using multi-scale multi-fractal detrended fluctuation analysis in mice: Roles of the autonomic nervous system and sinoatrial node

**DOI:** 10.3389/fphys.2022.970393

**Published:** 2022-09-27

**Authors:** Motahareh Moghtadaei, Tristan W. Dorey, Robert A. Rose

**Affiliations:** ^1^ Department of Cardiac Sciences, Cumming School of Medicine, Libin Cardiovascular Institute, University of Calgary, Calgary, AB, Canada; ^2^ Department of Physiology and Pharmacology, Cumming School of Medicine, Libin Cardiovascular Institute, University of Calgary, Calgary, AB, Canada

**Keywords:** heart rate, autonomic nervous system, heart rate variability, sinoatrial node, fractality, detrended fluctuation analysis

## Abstract

Nonlinear analyses of heart rate variability (HRV) can be used to quantify the unpredictability, fractal properties and complexity of heart rate. Fractality and its analysis provides valuable information about cardiovascular health. Multi-Scale Multi-Fractal Detrended Fluctuation Analysis (MSMFDFA) is a complexity-based algorithm that can be used to quantify the multi-fractal dynamics of the HRV time series through investigating characteristic exponents at different time scales. This method is applicable to short time series and it is robust to noise and nonstationarity. We have used MSMFDFA, which enables assessment of HRV in the frequency ranges encompassing the very-low frequency and ultra-low frequency bands, to jointly assess multi-scale and multi-fractal dynamics of HRV signals obtained from telemetric ECG recordings in wildtype mice at baseline and after autonomic nervous system (ANS) blockade, from electrograms recorded from isolated atrial preparations and from spontaneous action potential recordings in isolated sinoatrial node myocytes. Data demonstrate that the fractal profile of the intrinsic heart rate is significantly different from the baseline heart rate *in vivo*, and it is also altered after ANS blockade at specific scales and fractal order domains. For beating rate in isolated atrial preparations and intrinsic heart rate *in vivo*, the average fractal structure of the HRV increased and multi-fractality strength decreased. These data demonstrate that fractal properties of the HRV depend on both ANS activity and intrinsic sinoatrial node function and that assessing multi-fractality at different time scales is an effective approach for HRV assessment.

## Introduction

Heart rate (HR), a critical measure of cardiac performance, is determined by the intrinsic properties of the sinoatrial node (SAN) and modulated by the autonomic nervous system (ANS) (Mangoni and Nargeot, 2008; Lakatta et al., 2010; Macdonald et al., 2020). Specifically, intrinsic HR is determined by the rate of spontaneous action potential (AP) firing in SAN myocytes while the sympathetic (SNS) and parasympathetic (PNS) nervous systems increase and decrease SAN AP firing, respectively. Heart rate variability (HRV), which describes the beat-to-beat variation in HR (i.e. variation in the R-R interval on the electrocardiogram), is understood to be robust in a healthy cardiovascular system (Billman, 2011; Yaniv et al., 2014; Yaniv et al., 2016). Consistent with this concept, reductions in HRV are associated with worse prognosis in a number of conditions and disease states (Hillebrand et al., 2013). HRV is recognized to arise from changes in the activity of the ANS (i.e. changes in sympatho-vagal balance) as well as alterations in the intrinsic properties of the SAN (i.e. changes in intrinsic HR) (Papaioannou et al., 2013; Yaniv et al., 2014; Behar et al., 2018; Dorey et al., 2020; Rosenberg et al., 2020). HRV is conventionally assessed through spectral and/or time domain analyses, which are well-established approaches that provide important insight into the roles of the autonomic nervous system (ANS) and intrinsic sinoatrial node (SAN) function in HRV (Billman, 2011; Shaffer and Ginsberg, 2017; Moen et al., 2019). HRV can also be assessed using a number of nonlinear approaches such as sample entropy (Richman and Moorman, 2000), Poincaré plots with analysis of standard deviations (Guzik et al., 2007), long range RR interval turbulence (Lombardi and Stein, 2011) and heart rate fragmentation (Costa et al., 2017).

While each of the above traditional and nonlinear approaches can provide important information on HRV in different conditions, it is important to recognize that the dynamic cardiovascular system is embedded in a highly complex fractal structure of interacting sub-systems such as fractal vascular and neural networks, humoral pathways and the SAN itself. This dissipative system is therefore best described as one that exists in a dynamic self-organized state, with nonlinear and fractal characteristics, that can preserve homeostasis (Dini et al., 2012; Castiglioni et al., 2018). HR and HRV represent complex signals within the cardiovascular system that provide important information on this process. The natural variability in HR is not characterized by any particular time scale meaning that HR, like other physiological signals, is fractal with numerous nonlinear features that are not quantifiable by traditional spectral analysis (Ivanov et al., 1999). As a result, kinetic fractals of the RR interval can be indicative of their self-similarity or their long-range correlation within RR interval time series. Accordingly, analysis of the fractal nature of HRV requires the use of distinct algorithms that facilitate the study of nonlinear parameters that describe this complex regulation of heart rate as not all information contained in the HRV signal is captured by more traditional methods (Sassi et al., 2015). Reliable identification and validation of the fractal components of HRV can reveal alterations in cardiovascular regulatory mechanisms that could provide accurate insight into the physiology of HR regulation and help determine the risk of poor clinical cardiovascular outcomes.

Detrended Fluctuation Analysis (DFA) is an established algorithm that can be used to quantify the nonlinear fractal dynamics of the HRV time series by quantifying characteristic exponents that describe the scaling of the signal’s fluctuations (Makowiec et al., 2006; Gao et al., 2013; Constantinescu et al., 2018; Mizobuchi et al., 2021). Adaptive Fractal Analysis (AFA) is another nonlinear method that quantifies fractal geometry. Although AFA and DFA techniques are similar, AFA requires creation of a globally smooth trend signal by patching together local polynomial fits to the time series. DFA on the other hand, relies on discontinuous, piece-wise linear fits, which provides the rationale to progress from DFA to Multi-Fractal DFA (MFDFA) for multi-fractal analysis of the HR time series rather than its mono-fractal analysis (Maity et al., 2015). When compared to other nonlinear fractal analysis methods, a primary advantage of DFA is that it permits the detection of intrinsic self-similarity embedded in the seemingly nonstationary HR time series and, because it eliminates the local trends in the signal, it avoids false detection of artificial self-similarity in extrinsic trends. Other advantages of DFA are that it is applicable to short time series, it requires fewer data points compared to time and frequency domain analysis methods, and it is robust to noise and nonstationarity (Gieraltowski et al., 2012). A limitation of the basic DFA algorithm is that it only provides a mono-fractal description (Ivanov et al., 1999; Sassi et al., 2009); whereas HR is recognized to be a multi-fractal time series that shows self-similarity at different scales and different amplitudes with fractal properties that vary from point to point along the time series (Gieraltowski et al., 2012; Xia et al., 2013). As a result, a spectrum of scale exponents is more appropriate for assessing the multi-scale structure of HR changes and for investigating the effects of ANS activity and intrinsic SAN function on complex HR dynamics.

Thus, to more accurately assess non-linear HRV properties, we have used Multi-Scale Multi-Fractal Detrended Fluctuation Analysis (MSMFDFA) (Gieraltowski et al., 2012; Xia et al., 2013; Castiglioni et al., 2018) as a complexity-based method to jointly assess multi-scale and multi-fractal dynamics of HR in healthy wildtype mice in the ultra-low and very-low frequency bands (0.0024–0.33 Hz). The ultra-low frequency is thought to be driven by circadian rhythms and there is disagreement about the contributions of the SNS and PNS to ultra-low frequency oscillations in HR (Shaffer et al., 2014; Shaffer and Ginsberg, 2017). The very-low frequency band is driven by sympatho-vagal balance and the PNS may contribute to this band more than the SNS (Shaffer and Ginsberg, 2017). The very-low frequency band is strongly associated with all-cause mortality as well as arrhythmic death and is therefore thought to be fundamental to overall health (Shaffer and Ginsberg, 2017). The lower range of the ultra-low frequency band, in particular, is not typically captured in traditional frequency domain analysis; therefore, the MSMFDFA approach can provide novel information not possible with other approaches.

While some studies have used multi-scale DFA analysis in humans (Castiglioni et al., 2017; Castiglioni and Faini, 2019) and in isolated cardiac myocytes (chick embryonic myocytes) (Ahammer et al., 2013), a rigorous application of MSMFDFA across several levels of organization (i.e. from *in vivo* to isolated SAN myocytes) has not been reported. It is essential to do this because it is recognized that HRV can arise from changes in ANS activity as well as changes in intrinsic SAN function (Yaniv et al., 2013; Yaniv et al., 2016; Dorey et al., 2020; Dorey et al., 2021). Accordingly, we have applied MSMFDFA analysis to measurements of heart rate/beating rate conducted at multiple levels of organization including in unrestrained mice *in vivo* (baseline and in the presence of ANS antagonists), in isolated atrial preparations (containing the intact SAN, but devoid of neural inputs) and in isolated SAN myocytes. These studies enable us to accurately assess the impacts of the ANS and intrinsic SAN function on complex HR dynamics across multiple scales in the ultra-low and very-low frequency ranges using non-linear MSMFDFA analysis.

## Materials and methods

### Mice

This study used adult male wildtype C57Bl/6 mice between the ages of 10 and 15 weeks. All experimental procedures used in this study followed the Canadian Council on Animal Care guidelines and were approved by the University of Calgary Animal Care and Use Committee. Mice were housed in groups of 3-5 per cage using Tecniplast Green Line GM500 cages with 500 cm^2^ of floor space and provided with enrichment items (nesting material, houses) in the cages. Mice were provided with standard rodent chow (LabDiet 5062) and water *ad libitum*. Mice were kept on a 12:12 h light:dark cycle. Temperature in the room was maintained at 21–22°C and humidity was 32–38%. These environmental and housing conditions were monitored daily and maintained throughout the study.

### Telemetry ECG recording

To investigate HRV *in vivo*, we used telemetric ECG recordings as we have described (Moghtadaei et al., 2017; Dorey et al., 2020; Dorey et al., 2022). ECGs and activity were monitored in awake, freely moving mice using subcutaneously implanted telemetric transmitters (HD-X11, Data Sciences International). After 7 days of recovery following transmitter insertion, telemetric ECG recordings and activity levels were acquired continuously for 48 h. After the baseline recording period was completed, the effects of ANS blockade were investigated by intraperitoneal injection of the β-adrenergic receptor (β-AR) antagonist propranolol hydrochloride (10 mg/kg) and the muscarinic (M2) receptor antagonist atropine sulfate (10 mg/kg), alone or in combination, at approximately the same time each day (9:00–11:00 a.m.). A minimum of 24 h was given between injections. After drug injection, the ECG was recorded continuously for 1 h. ECG data acquisition, ECG filtering and R-wave detection was done using Ponemah software (Data Sciences International).

### Electrogram recordings in isolated atrial preparations

Intact atrial preparations containing the SAN, but devoid of autonomic innervation, were prepared as we have described previously (Dorey et al., 2020; Dorey et al., 2021). Beating atrial preparations were superfused continuously with Krebs solution (37°C) containing (in mM): 118 NaCl, 4.7 KCl, 1.2 KH_2_PO_4_, 12.2 MgSO_4_, 1 CaCl_2_, 25 NaHCO_3_, 11 glucose. This Krebs solution was bubbled with 95% O_2_/5% CO_2_ in order to maintain a pH of 7.4. After the atrial preparation was equilibrated for at least 30 min, electrograms were measured continuously using needle electrodes (Grass Technologies) placed in each atrial appendage. Electrograms were acquired using a Powerlab 26T (AD Instruments).

### Action potential recordings in isolated sinoatrial node myocytes

SAN myocytes were isolated using procedures we have described previously (Rose et al., 2004; Mackasey et al., 2018). Spontaneous action potentials (APs) were recorded using the perforated patch-clamp technique on single SAN myocytes. For recording APs the recording chamber was superfused with a normal Tyrode’s solution containing (in mM): 140 NaCl, 5 KCl, 1 MgCl_2_, 1 CaCl_2_, 10 HEPES, and 5 glucose, with pH adjusted to 7.4 with NaOH. The pipette filling solution contained (in mM) 135 KCl, 0.1 CaCl_2_, 1 MgCl_2_, 5 NaCl, 10 EGTA, 4 Mg-ATP, 6.6 Na-phosphocreatine, 0.3 Na-GTP and 10 HEPES, with pH adjusted to 7.2 with KOH. Amphotericin B (200 μg/ml) was added to this pipette solution to record APs with the perforated patch clamp technique. APs were recorded at room temperature (22–23°C).

### Analysis of ECG recordings and heart rate variability

The ECG signal was first divided into low and high activity phases based on activity measurements from telemeters. The ECG signals at high and low activity were each divided into 2 h segments and each 2 h segment was divided into 2000 beat segments for the fractal analysis. For each activity level, five separate 2 h segments were analyzed and averaged. For the signals recorded from isolated atrial preparations and isolated SAN myocytes, peak detection was performed on the electrogram recordings in atrial preparation or spontaneous APs in SAN myocytes, respectively. Segments with 2000 and 1,000 beats from atrial preparations and SAN myocytes were selected for the fractal analysis respectively. The MSMFDFA algorithm is robust to noise and non-stationarity; therefore, it was not necessary to identify and remove sinus pauses and ectopic activity in the tachogram.

### Time and frequency domain HRV analysis

Time and frequency domain metrics were analyzed using customized software written in MATLAB (MathWorks, Natick, Massachusetts) as we have described previously (Moghtadaei et al., 2017; Dorey et al., 2021). HRV was assessed using time and frequency domain analysis from ECG recordings *in vivo*. Stationary NN interval time series of at least 5 min in duration were used for time domain analysis. Each episode was examined to ensure a stationary and stable sinus rhythm with no trend or periodic fluctuations. Next, R wave detection was performed, and RR interval time series were obtained. In isolated atrial preparations, NN intervals were defined as the interval between peaks on the electrogram recordings in atrial preparations. The time domain parameters we are reporting include the standard deviation of all normal RR intervals (SDNN, in ms) and the root mean square differences between successive RR intervals (RMSSD, in ms).

To correct for the influence of HR on SDNN, we plotted SDNN as a function of HR for all baseline data and fitted these data with an exponential function, which was then used to generate the following equation to correct for HR and produce corrected SDNN (cSDNN).

$$cSDNN=\frac{SDNN}{e^{-0.004}\times HR}$$ (1)

For frequency domain analysis, each of the low and high activity phases were divided into 2 min episodes. These time frames were chosen in order to ensure that each episode contained at least 1,024 data points (R waves). Similar to the time domain analysis, each episode was manually examined to ensure a stationary and stable sinus rhythm, which is required for performing fast Fourier transforms (see below). Next, R wave detection was performed, and the RR interval time series were generated. Linear trends and drift were removed from the signal to reveal the HRV in the data. In the present study, we have used Welch’s method to characterize the frequency content of the signal (i.e. to estimate the power of the signal at different frequencies). In Welch’s method, the signal is broken into overlapping segments to reduce noise in the frequency spectrum. Then, the segments are windowed to reduce spectral leakage. The periodogram of each windowed segment is calculated using the Fourier transform. Finally, the periodograms are averaged to make a single frequency spectrum. In the present study, we have used 50% overlapping and the Hamming window for the spectral density estimation. The total power of each periodogram was measured as a total index of HRV, which determines the integral of total variability over the entire frequency range. Then, the high frequency (HF) and low frequency (LF) components were extracted. The HF component of HRV (1.5–5 Hz) is predominantly mediated by the phasic activity of the parasympathetic nervous system. The LF oscillations of HR (0.1–1.5 Hz) are regulated by both the sympathetic and parasympathetic nervous systems; however, the tonic sympathetic component is dominant.

### MSMFDFA algorithm

The MSMFDFA algorithm used in this study is based on the conventional DFA algorithm. Given an R-R interval time series x(j) with mean $\bar{x}$ , evaluated over *N* consecutive heart beats (1 ≤ *j* ≤ *N*), its cumulative sum is calculated as Eq. 2.

$$y\left(i\right)=\sum_{j=1}^{i}\left(x\left(j\right)-\bar{x}\right),\;i=1,2,\ldots ,N$$ (2)

The y(i) signal is then segmented into $B_{S}$ 70% overlapping blocks of size s, resulting in $y_{s}\left(b\right),\;b=1,\;2,\ldots ,\;B_{s}$ . A short segment of $N-sB_{s}$ data at the end of the series is not included in the analysis. 70% overlap was used to save computation time compared to maximal overlap, while keeping the estimator variance low compared to no overlap, with the final result being very similar to the maximal overlap case.

The first order trend of each segment $y_{s}\left(b\right)$ is calculated as its least square fit $Y_{s}\left(b\right),\;b=1,\;2,\ldots ,B_{s}$ . Then the variance of the detrended signal $\left(y_{s}\left(b\right)-Y_{s}\left(b\right)\right)$ is calculated using Eq. 3.

$$F\left(s\right)=\sqrt{\frac{1}{B_{s}}\sum_{b=1}^{B_{s}}{\left(y_{s}\left(b\right)-Y_{s}\left(b\right)\right)}^{2}}$$ (3)

The variance, F(s) , is a function of scale, s, and this procedure is repeated over a range of different scales (i.e. from 3 to 415 s). Next, the q order fluctuation function is calculated using Eq. 4.

$$F_{q}\left(s\right)=\{\begin{array}{cc} {\frac{1}{B_{s}}\sum_{b=1}^{B_{s}}{\left(F\left(s\right)\right)}^{q}}^{1/q} & q\neq 0\\ e^{\frac{1}{2B_{s}}\sum_{b=1}^{B_{s}}\ln F\left(s\right)} & q=0 \end{array}$$ (4)

The slope of this surface $\left(\alpha_{q}\left(s\right)\right)$ is the q-order generalized Hurst exponent, also known as MSMFDFA scale exponent (Castiglioni et al., 2011). In general, the Hurst exponent is known to be less than one for fractional Gaussian noise, and greater than one for fractional Brownian motion (Ihlen, 2012). If x(j) has fractal characteristics, $F_{q}\left(s\right)$ increases as a power-law function, i.e. $F_{q}\left(s\right)\propto s^{\alpha_{q}\left(s\right)}$ with $\alpha_{q}\left(s\right)>1$ . MSMFDFA scale exponents provide important information about the HRV signal. Specifically:

1- If the time series x(j) has long-range fractals, $F_{q}\left(s\right)$ increases as a power-law $\left(F_{q}\left(s\right)\propto s^{\alpha_{q}\left(s\right)},\;\alpha_{q}\left(s\right)>1\right)$ for large values of *s.* Similarly, if x(j) has short-range fractals, $\alpha_{q}\left(s\right)>1$ for small values of *s.*
2- If x(j) is mono-fractal then $F_{q}\left(s\right)$ increases as a power of *s* for any choice of the parameter *q*( $F_{q}\left(s\right)\alpha s^{\alpha_{q}\left(s\right)}$ ), and α(s) is independent of *q.* If x(j) is a multi-fractal time series, its small and large fluctuations scale differently and there will be a significant dependence of $\alpha_{q}\left(s\right)$ on *q.* In this case, $\alpha_{q}\left(s\right)$ mainly reflects the fractal components with larger and smaller amplitudes if *q* > 0 and *q* < 0 respectively (Kantelhardt et al., 2002).

In this study, both scale (*s*) and order (*q*) dependencies of the fractal structures in the HRV signal were quantified. The analysis was performed in the beat domain and then each scale coefficient was associated with its temporal scale, by mapping the beat domain into the time domain. This step is necessary for comparing different conditions with different average HR values.

The multi-fractality was also quantified as a function of scale using the multi fractal index (MFI(s)) . Specifically, for each scale *s*, the standard deviation of all $\alpha_{q}\left(s\right)$ values estimated over the range −qr≤q≤qr , which is symmetric around 0 is calculated, and the MFI(s) was defined using Eq. 5 (Castiglioni et al., 2018).

$$MFI\left(s\right)=\frac{\sigma_{q}\left(\alpha_{q}\left(s\right)\right)}{2qr}$$ (5)

If instead of local slopes, for each value of q a single slope is calculated over the whole range of scales, then $F_{q}\left(s\right)\propto s^{A_{q}}$ , where

A_*q*_ is independent of scale. In this case A_*q*_ is related to the classical multi-fractal scaling exponents or Renyi index $\left(\tau_{q}\right)$ as given in Eq. 6.

$$\tau_{q}=qA_{q}-1$$ (6)

The multi-fractal spectrum $\left(D_{q}\right)$ is related to $\tau_{q}$ via a Legendre transform (Kantelhardt et al., 2002) given in Eqs 7, 8.

$$h_{q}=\tau_{q}^{\prime }$$ (7)

$$D_{q}=qh_{q}-\tau_{q}$$ (8)

Here, $D_{q}$ reflects the multi-fractal spectrum of the subset of series and its value reflects the fractal dimension with the singularity exponent of $h_{q}$ (Zhang et al., 2019) which is inversely related to singularity (i.e. bigger singularities have smaller $h_{q}$ values). The central tendency of $D_{q}$ represents the average fractal structure of the HRV. The deviation from average fractal structure for segments with large and small fluctuations is represented by the multi-fractal spectrum width $\left(h_{q_{max}}-h_{q_{min}}\right)$ . The width and shape of the multi-fractal spectrum classifies scale invariant structures of HRV time series. Larger multi-fractal spectrum width implies more unevenness of the time series distribution, and greater multi-fractal strength.

Spectra of MSMFDFA scale exponents, including the Hurst exponent, multi-fractal index, Renyi index, and multi-fractal spectrum were calculated for scales between 3 and 415 s, which can be considered as a qualitative measure of the power spectrum in the region of the ultra-low and very-low frequency bands, (i.e. at frequencies between 0.0024 and 0.33 Hz).

### Statistical analysis

All data are presented as means ± SEM. Data were analyzed using Student’s *t*-test, two-way repeated measures ANOVA test or two-way ANOVA with a Holm-Sidak posthoc test as indicated in each figure legend. *p* < 0.05 was considered significant.

## Results

### Time and frequency domain HRV analysis

Initially, standard time and frequency domain HRV metrics were calculated from ECGs, electrograms or spontaneous AP recordings for each experimental condition (Table 1). Specifically, *in vivo* analysis was performed in baseline low activity and baseline high activity conditions (activity was assessed telemetrically), as well as after application of the ANS blockers atropine, propranolol or both in combination. Time and frequency domain analysis was also done in isolated atrial preparations and in isolated SAN myocytes (Table 1). For mice the low frequency band was 0.1–1.5 Hz and the high frequency band was 1.5–5 Hz for traditional frequency domain analysis (Moghtadaei et al., 2017; Behar et al., 2018). The data in Table 1 demonstrate that values for time domain metrics (SDNN, cSDNN, RMSSD) and frequency domain metrics (total power, HF power, LF power) were comparable in each condition to those reported previously for mice *in vivo* (baseline and ANS blockade), as well as for isolated mouse atrial preparations and isolated mouse SAN myocytes (Moghtadaei et al., 2017; Dorey et al., 2020; Dorey et al., 2021; Dorey et al., 2022). In particular, ANS blockers reduced time and frequency domain measures of HRV *in vivo* as expected. Furthermore, time domain analysis demonstrates that variability was higher in isolated atrial preparations and isolated SAN myocytes compared to baseline low activity conditions *in vivo*, as demonstrated previously (Dorey et al., 2020; Dorey et al., 2021).

**TABLE 1:** Time and frequency domain analysis of HRV *in vivo*, in isolated atrial preparations and in isolated SAN myocytes.

Parameter	Baseline: low activity *in vivo*	Baseline: High activity *in vivo*	Atropine *in vivo*	Propranolol *in vivo*	Atropine and propranolol *in vivo*	Atrial preparation	SAN myocyte
Mean NN	126.1 ± 12.1	102.0 ± 4.7*	96.1 ± 5.7*	116.9 ± 4.0*	123.6 ± 7.8	153.9 ± 10.2*	410.7 ± 23.0*
SDNN	10.2 ± 2.4	8.6 ± 2.0	5.0 ± 1.1*	4.9 ± 0.90*	5.01 ± 1.5*	15.6 ± 3.8*	49.2 ± 8.2*
cSDNN	23.0 ± 4.7	23.4 ± 4.8	14.4 ± 2.6*	11.7 ± 2.3*	11.5 ± 3.3*	15.6 ± 3.7*	49.2 ± 8.2*
RMSSD	5.1 ± 1.3	4.6 ± 1.4	2.5 ± 0.8*	3.4 ± 0.7*	4.3 ± 1.7*	11.6 ± 2.7*	44.4 ± 8.9*
Total power	1.4 ± 0.9	1.4 ± 1.1	0.2 ± 0.1*	0.7 ± 0.3*	0.4 ± 0.2*	0.005 ± 0.002*	0.039 ± 0.01*
HF power	0.56 ± 0.3	0.53 ± 0.4	0.12 ± 0.1*	0.34 ± 0.2*	0.28 ± 0.1*	0.004 ± 0.002*	0.0003 ± 0.0001*
LF power	0.81 ± 0.6	0.83 ± 0.7	0.02 ± 0.01*	0.32 ± 0.2*	0.12 ± 0.1*	0.0013 ± 0.0006*	0.021 ± 0.008*

Mean NN, mean N-N interval; SDNN, standard deviation of all normal NN, intervals; cSDNN, corrected SDNN; RMSSD, root mean square of successive differences in NN, interval; HF, power, high frequency power; LF, power, low frequency power. Data are mean ± SEM; *n* = 7 mice for *in vivo* groups, *n* = 7 isolated atrial preparations, *n* = 6 SAN, myocytes. *p < 0.05 vs. baseline LA, by Student’s *t*-test.

### Effect of activity level on MSMFDFA

Next, nonlinear HRV was assessed using MSMFDFA, which enables assessment of HRV in the frequency range of 0.0024–0.33 Hz, encompassing the very-low frequency (0.0056–0.1 Hz) and ultra-low frequency bands (0–0.0056 Hz) (Behar et al., 2018).

**FIGURE 1:**
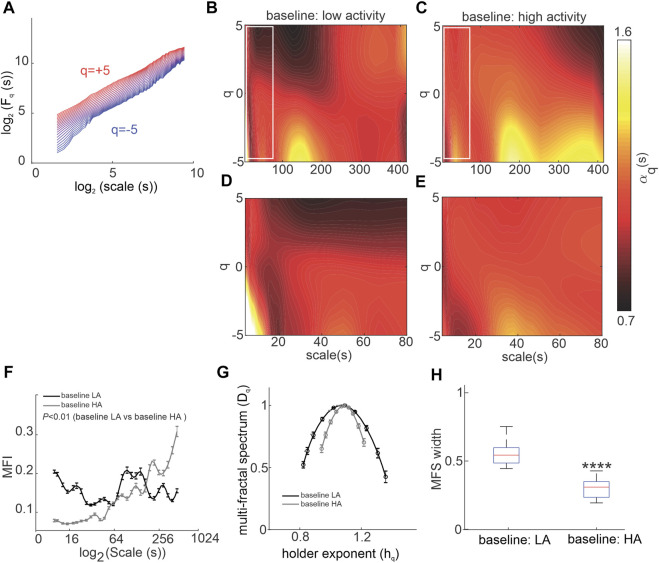
Multiscale multi-fractal detrended-fluctuation analysis of heart rate variability in wildtype mice *in vivo*. **(A)** Overall RMS $\left(F_{q}\left(s\right)\right)$ vs. scale for different orders (−5<q<5) where both $F_{q}$ and scale are represented in log_2_ coordinates. **(B, C)** Heat maps for MSMFDFA scale exponents $\left(\alpha_{q}\left(s\right)\right)$ as a function of order (q) and scale for −5<q<5 and 3s<scale<415s in baseline low activity **(B)** and baseline high activity **(C)**; data are an average of n = 7 mice, overlap = 70%. **(D, E)** Magnification of the regions marked with white rectangles in B and C for the HRV signal in baseline low activity **(D)** and baseline high activity **(E)** for 3s<scale<80s . **(F)** Multi-fractality index over 3s<scale<415s and qr=5 for baseline low activity and baseline high activity; *n* = 7 mice, data analyzed using two-way repeated measures ANOVA. **(G)** Multi-fractal spectrum of the HRV signal in baseline low activity and baseline high activity; *n* = 7 mice. **(H)** Multi-fractal spectrum width of the HRV signal in baseline low activity and baseline high activity; *n* = 7 mice; ****p = 1.8 × 10^−7^ vs. baseline low activity by Student’s *t*-test.

Nonlinear HRV was assessed from ECGs recorded in conscious, freely moving mice *in vivo*. In all groups and conditions, the generalized *q* dependent fluctuation function $F_{q}\left(s\right)$ of HRV increases as a function of scale confirming the underlying fractal nature of the HRV signal (Figure 1A). Scale (i.e. time scale) describes the time range (in seconds) in which the fractals are found and which were analyzed (Castiglioni and Faini, 2019). The existence of local deviations from the overall linear slope in the log-log plot (Figure 1A) indicates the dependence of $F_{q}\left(s\right)$ on the *q* order, confirming the multi-fractality of the HRV signal. Thus, order is used to extract and quantify the specific fractal amplitude. Large amplitude fluctuations that have fractal characteristics are found when positive orders of the fluctuation function are calculated while small amplitude fluctuations with fractal characteristics are found when negative orders of the fluctuation function are calculated (Castiglioni and Faini, 2019).

The low and high-activity phases were separated based on activity measured from telemeters. MSMFDFA parameters were then measured for ECG in phases of high and low activity. In baseline conditions for most scales, $\alpha_{q}\left(s\right)>1$ for *q < 0* indicating the existence of low-amplitude fractals in the signal; however, the scale dependence is affected by the activity level (Figures 1B,C). Specifically, the long-range (scale>180 s) low amplitude (q < 0) fractals are stronger in high activity conditions compared to low activity conditions (Figures 1B,C). The mid-range (100 s < scale<180 s; Figures 1B,C) and short-range (scale<15s; Figures 1D,E) low amplitude fractals, on the other hand, are stronger during low activity. Overall, analysis of the multi fractal index shows larger variability in scale exponents for baseline low activity episodes for scales less than 180 s and for high activity episodes for scales greater than 180 s (Figure 1F).

During high activity episodes, when SNS activity is increased, the average fractal structure of the HRV signal was increased compared to low activity, which is evident from the shift in the central tendency of the multi-fractal spectrum of the HRV signal towards smaller singularities (i.e. larger *h*_*q*_ values; Figure 1G). Furthermore, weaker multi-fractality during high activity episodes is evident from the smaller multi-fractal spectrum width (Figure 1H).

### ANS modulation of MSMFDFA on HRV *in vivo*

**FIGURE 2:**
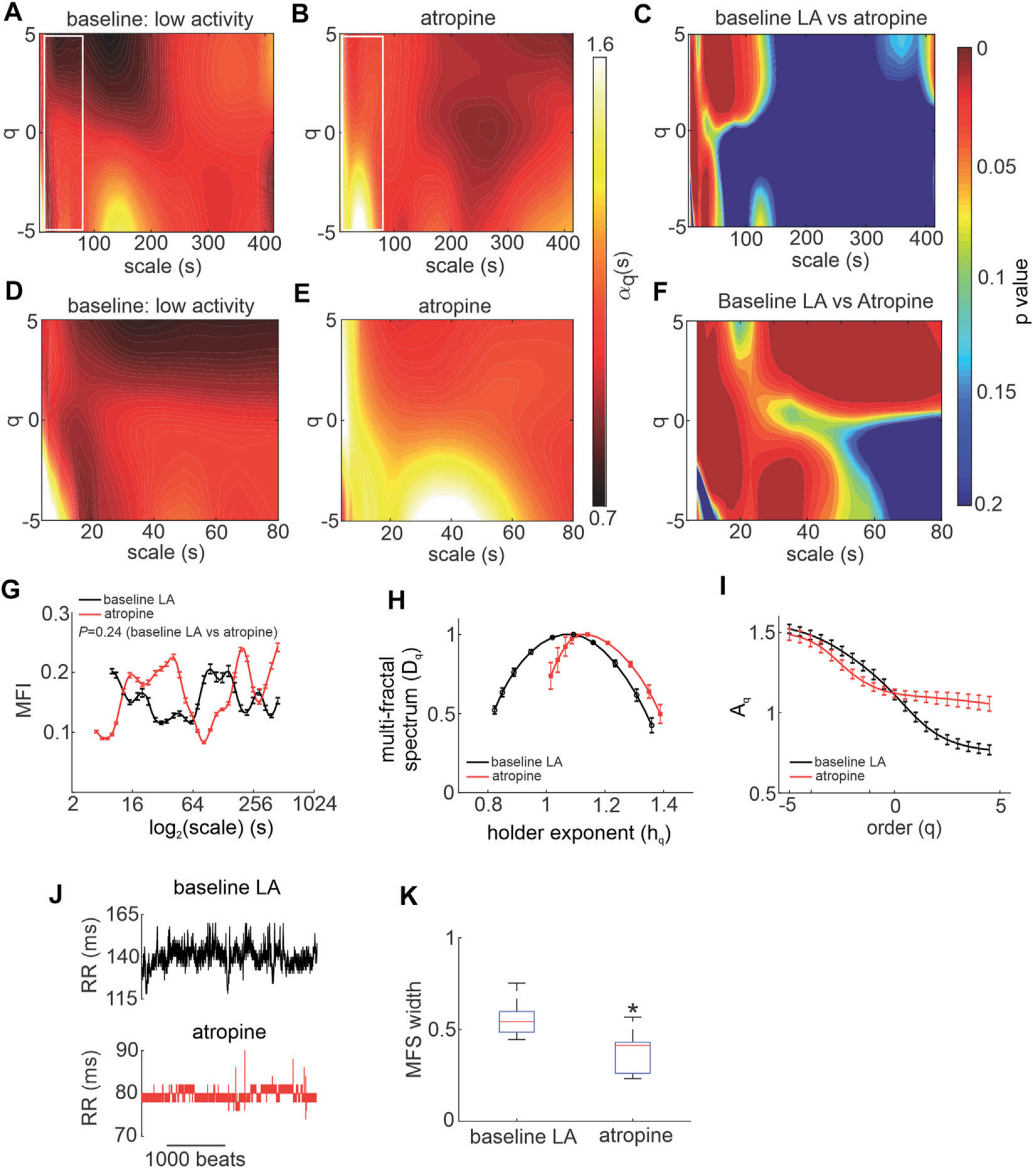
Effects of atropine on MSMFDFA of heart rate variability in wildtype mice in vivo. **(A, B)** Heat maps for MSMFDFA scale exponents $\left(\alpha_{q}\left(s\right)\right)$ as a function of order (q) and scale for −5<q<5 and 3s<scale<415s in baseline low activity **(A)** and after intraperitoneal injection of atropine **(B)**. **(C)** Statistical comparison of the MSMFDFA scale exponent of the HRV signal in baseline low activity vs. atropine using two tailed Student’s *t*-test for −5<q<5 and 3s<scale<415s ; *n* = 7 mice. **(D,E)** Magnification of the regions marked with white rectangles in A and B for the HRV signal in baseline low activity **(D)** and after application of atropine **(E)** for 3s<scale<80s . **(F)** Statistical comparison of the MSMFDFA scale exponent of the HRV signal in baseline low activity vs. atropine using two tailed Student’s *t*-test for −5<q<5 and 3s<scale<80s ; *n* = 7 mice. **(G)** Multi-fractality index over 3s<scale<415s and qr=5 for baseline low activity and atropine, *n* = 7 mice, data analyzed using two-way repeated measures ANOVA. **(H)** Multi-fractal spectrum of the HRV signal in baseline low activity and after application of atropine; *n* = 7 mice. **(I)** Leveling of the q-order multi-fractal exponent of the HRV signal for positive *q* values after intraperitoneal injection of atropine; *n* = 7 mice. **(J)** Representative tachograms in baseline LA conditions and after application of atropine. **(K)** Multi-fractal spectrum width of the HRV signal in baseline low activity and after application of atropine; *n* = 7 mice; *p = 0.018 vs. baseline low activity by Student’s *t*-test.

To directly investigate the effects of ANS activity on MSMFDFA of HRV, mice were given ANS blockers beginning with atropine to block the PNS (Figure 2). Atropine substantially altered the spectrum of HRV scale exponents compared to baseline low activity conditions as shown in scale exponent heat maps (Figures 2A–C). Atropine eliminated mid-range low amplitude fractals at 100 s < scales<200 s and q < 0 and decreased $\alpha_{q}\left(s\right)$ to values less than one in this range (Figures 2A,B). At scales smaller than 80 s, atropine increased $\alpha_{q}\left(s\right)$ values above one for *q > 0* (Figures 2B,C). Atropine also increased $\alpha_{q}\left(s\right)$ at 15 s < scales<50 s and q < 0, as well as at scales<15 s and q > 0, and decreased $\alpha_{q}\left(s\right)$ to values below one for scales<10 s and q < -4 (Figures 2D–F).

Consistent with these heat maps, analysis of the multi fractal index following atropine injection shows there is larger variability in scale exponents of the HRV signal during PNS blockade for the majority of scales from 3 to 415 s, except for scales<10 and 100 s < scales<200 s when compared to baseline low activity (Figure 2G).

The multi-fractal spectrum of HRV after atropine injection was asymmetric with left tail truncation (Figure 2H) that originated from a leveling of the q-order multi-fractal exponent for positive *q* values (Figure 2I) indicating that the q-order DFA is insensitive to local fluctuations with large magnitudes. Representative tachograms for baseline low activity and atropine injection confirm that the variability of RR intervals was smaller, and lacked large amplitude local fluctuations, after atropine injection (Figure 2J).

In addition, the fractal structure of the HRV signal was increased after injection of atropine as illustrated by a shift in the central tendency of the multi-fractal spectrum of the HRV signal towards smaller singularities (larger *h*_*q*_ values; Figure 2H). This occurred in association with a reduction in multi-fractality strength as shown by a smaller multi-fractal spectrum width in the presence of atropine (Figure 2K).

**FIGURE 3:**
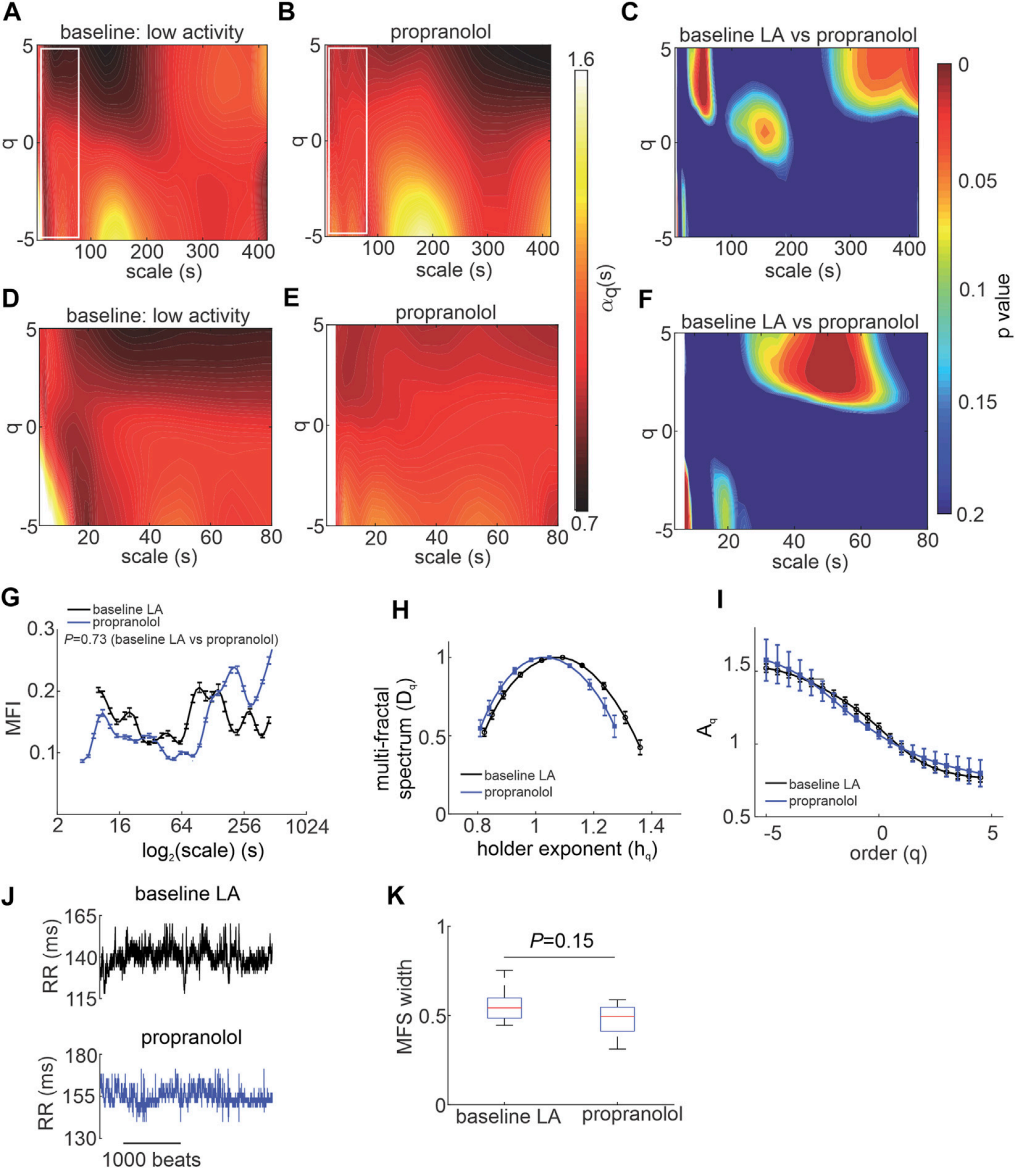
Effects of propranolol on MSMFDFA of heart rate variability in wildtype mice in vivo. **(A, B)** Heat maps for MSMFDFA scale exponents $\left(\alpha_{q}\left(s\right)\right)$ as a function of order (q) and scale for −5<q<5 and 3s<scale<415s in baseline low activity **(A)** and after intraperitoneal injection of propranolol **(B)**. **(C)** Statistical comparison of the of MSMFDFA scale exponent of the HRV signal in baseline low activity vs. propranolol using two tailed Student’s *t*-test for −5<q<5 and 3s<scale<415s ; *n* = 7 mice. **(D,E)** Magnification of the regions marked with white rectangles in A and B for the HRV signal in baseline low activity **(D)** and after application of propranolol **(E)** for 3s<scale<80s . **(F)** Statistical comparison of the MSMFDFA scale exponent of the HRV signal in baseline low activity vs. propranolol using two tailed Student’s *t*-test for −5<q<5 and 3s<scale<80s ; *n* = 7 mice. **(G)** Multi-fractality index over 3s<scale<415s and qr=5 for baseline low activity and propranolol; *n* = 7 mice, data analyzed using two-way repeated measures ANOVA. **(H)** Multi-fractal spectrum of the HRV signal in baseline low activity and after application of propranolol; *n* = 7 mice. **(I)** q-order multi-fractal exponent of the HRV signal as a function of q value after application of propranolol; *n* = 7 mice. **(J)** Representative tachograms in baseline LA conditions and after application of propranolol **(K)** multi-fractal spectrum width of HRV signal in baseline low activity and after propranolol; *n* = 7 mice, data analyzed by Student’s *t*-test.

Next, the effects of SNS blockade on MSMFDFA of HRV were investigated by injecting mice with propranolol (Figure 3). Except for few regions, the overall MSMFDFA spectrum remained similar to baseline low activity conditions after propranolol injection. Heat maps of scale exponents show that propranolol decreased $\alpha_{q}\left(s\right)$ at 310 s < scales and q > 0 with values remaining less than 1 (Figures 3A–C) and increased $\alpha_{q}\left(s\right)$ at 30 s < scales<60 s and q > 0 without exceeding 1 (Figures 3D–F). Propranolol also decreased $\alpha_{q}\left(s\right)$ at short scales (<10 s) for q < 0 to values less than 1 (Figures 3D–F) indicating that blocking the SNS eliminates low amplitude short-range fractals.

Overall, the multi fractal index was not different when comparing propranolol to baseline low activity conditions despite values being lower on average for scales<127 s after application of propranolol (Figure 3G). The multi-fractal spectrum of the HRV signal after injection of propranolol remained symmetric and was similar to baseline low activity conditions with no differences in central tendency (Figure 3H). Consistent with this, the q-order multi-fractal exponent was similar between baseline LA and propranolol (Figure 3I) and representative tachograms show similar patterns of fluctuation between these conditions (Figure 3J). Finally, the multi-fractal spectrum width was not different between baseline LA and propranolol (Figure 3K). This indicates that propranolol did not change the fractal structure of the HRV signal or its multi-fractality strength.

### Intrinsic MSMFDFA of HRV *in vivo* and *ex vivo*

**FIGURE 4:**
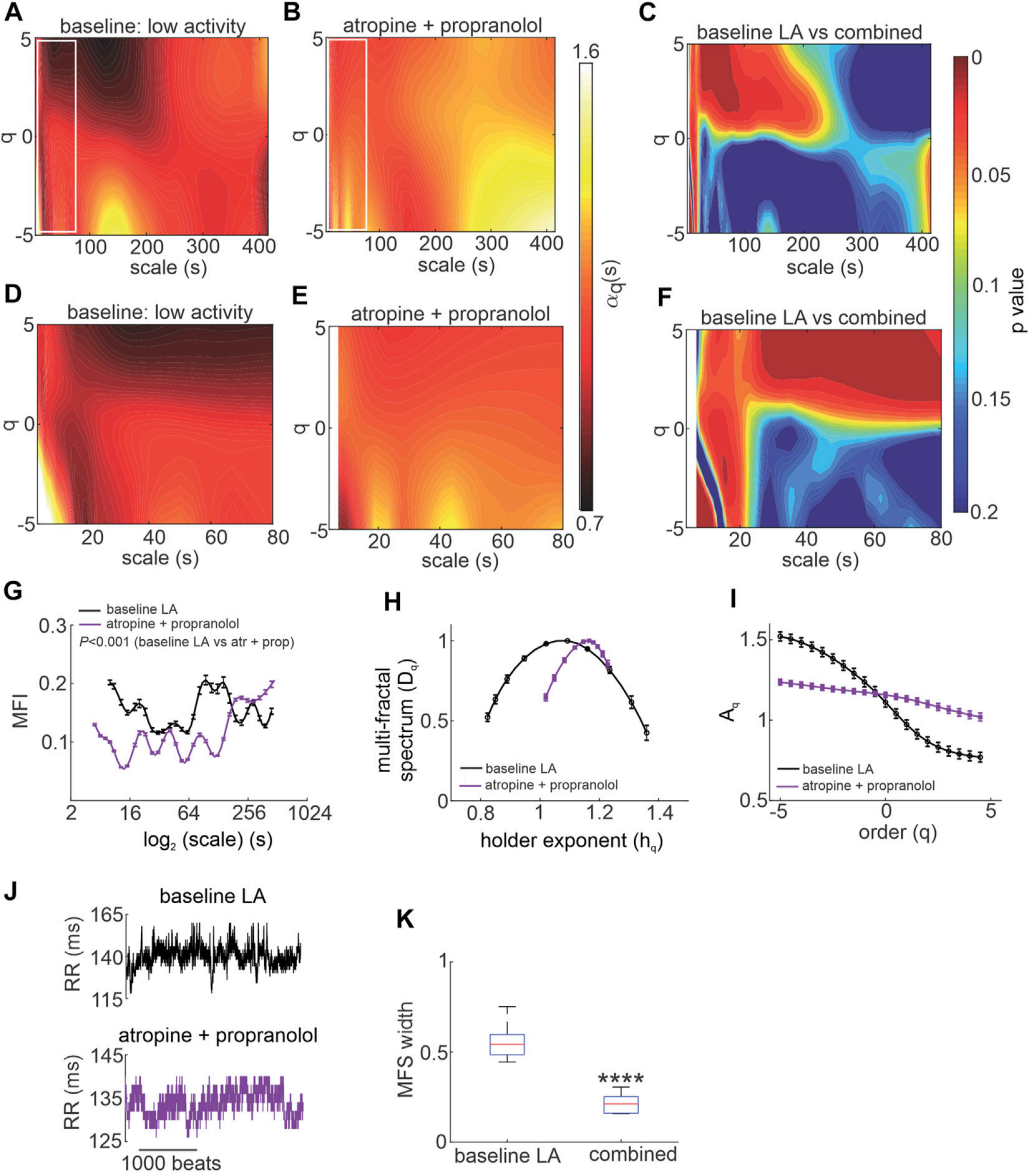
Effects of autonomic nervous system blockade on MSMFDFA of heart rate variability in wildtype mice in vivo. **(A, B)** Heat maps for MSMFDFA scale exponents $\left(\alpha_{q}\left(s\right)\right)$ as a function of order (q) and scale for −5<q<5 and 3s<scale<415s in baseline low activity **(A)** and after combined intraperitoneal injection of atropine + propranolol **(B)**. **(C)** Statistical comparison of the of MSMFDFA scale exponent of the HRV signal in baseline low activity vs. atropine + propranolol using two tailed Student’s *t*-test for −5<q<5 and 3s<scale<415s ; *n* = 7 mice. **(D, E)** Magnification of the regions marked with white rectangles in A and B for the HRV signal in baseline low activity **(D)** after application of atropine + propranolol **(E)** for 3s<scale<80s . **(F)** Statistical comparison of the MSMFDFA scale exponent of the HRV signal in baseline low activity and after application of atropine and propranolol using two tailed Student’s *t*-test for −5<q<5 and 3s<scale<80s ; *n* = 7 mice **(G)** multi-fractality index over 3s<scale<415s and qr=5 for baseline low activity and atropine + propranolol; *n* = 7 mice; data analyzed by two-way repeated measures ANOVA. **(H)** Multi-fractal spectrum of the HRV signal in baseline low activity and after atropine + propranolol; *n* = 7 mice. **(I)** Leveling of the q-order multi-fractal exponent of the HRV signal for negative *q* values after application of atropine + propranolol; *n* = 7 mice. **(J)** Representative tachograms in baseline LA conditions and after application of atropine + propranolol. **(K)** Multi-fractal spectrum width of the HRV signal in baseline low activity and after atropine + propranolol; *n* = 7 mice; ****p = 8.6 × 10^−8^ vs. baseline low activity by Student’s *t*-test.

HRV is determined in part by intrinsic SAN function. Accordingly, intrinsic MSMFDFA of HRV was investigated in the presence of ANS blockade *in vivo*. Combined injection of atropine and propranolol altered the MSMFDFA scale exponents throughout the measured parameter space of 3 s < scales<415 s and -5<q < 5 compared to baseline low activity such that $\alpha_{q}\left(s\right)$ was always greater than one (Figures 4B,C). This indicates that the HRV signal became fractal on different ranges and orders after ANS blockade. The only exception to this occurred at scales<15 s and q < -3 where the short-range, low amplitude fractals that were present and strong in baseline low activity as a marker of the intact ANS (Figure 4D) are eliminated after injection of atropine and propranolol (Figures 4E,F).

Analysis of the multi fractal index after ANS blockade shows that there is less variability in scale exponents of the HRV signal for 3 s < scales<180 s compared to baseline low activity conditions (Figure 4G). The multi-fractal spectrum after injection of atropine and propranolol was asymmetric with a right tail truncation (Figure 4H) in association with a leveling of the q-order multi-fractal exponent for negative *q* values (Figure 4I). This indicates that the q-order DFA is insensitive to the local fluctuations with small magnitudes after ANS blockade. In agreement with this, representative tachograms demonstrate less low amplitude local variability after injection of atropine and propranolol compared to baseline low activity conditions (Figure 4J).

The presence of $\alpha_{q}\left(s\right)>1$ for most of the measurement region after ANS blockade is due to an increase in the average fractal structure of the HRV. Consequently, the central tendency of the multi-fractal spectrum of the HRV signal shifts towards smaller singularities (larger *h*_*q*_ values; Figure 4H). Furthermore, because the MSMFDFA spectrum became more uniform with less variability, the multi-fractal spectrum width was significantly shorter after combined injection of atropine and propranolol (Figure 4K) indicating a reduction in multi-fractality strength after ANS blockade.

**FIGURE 5:**
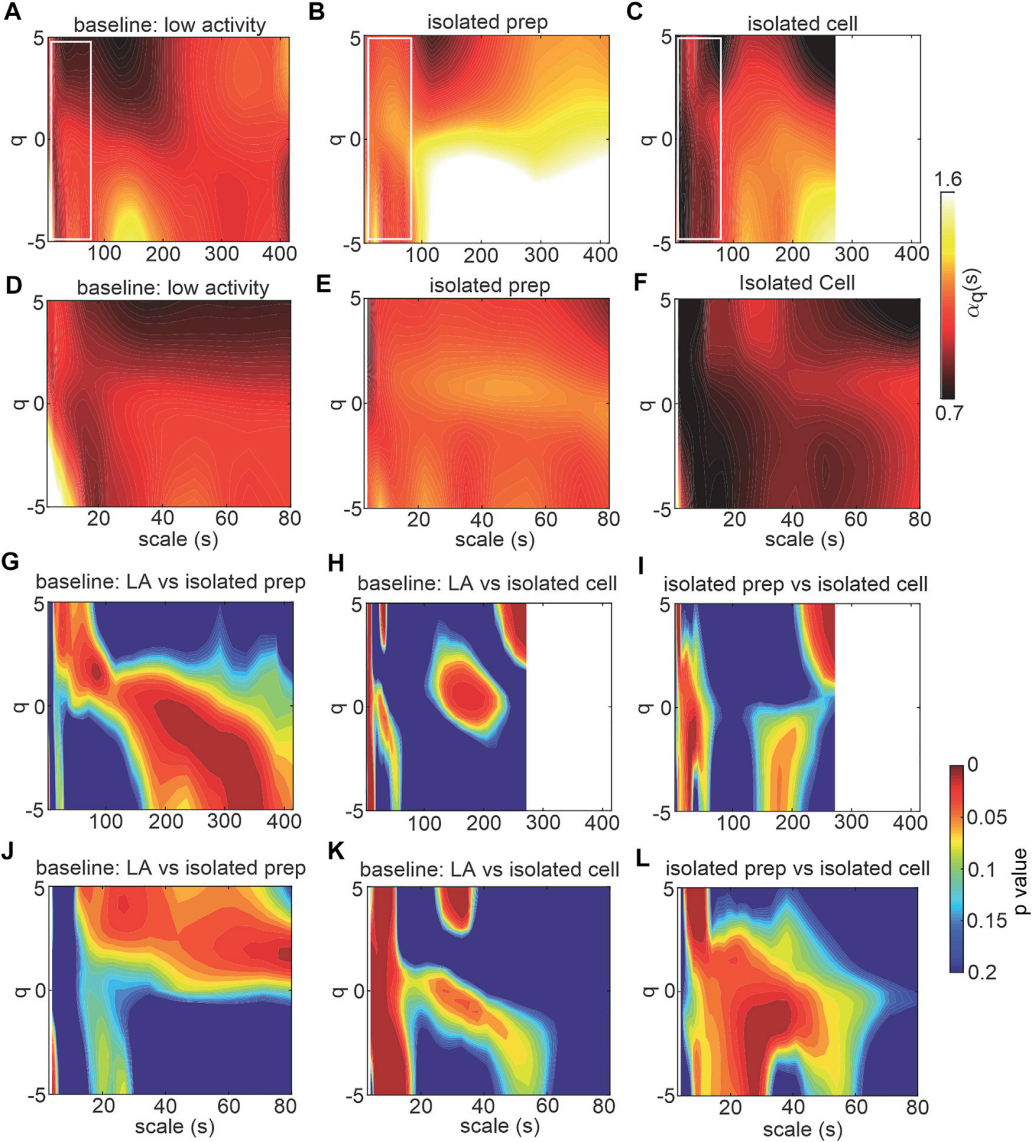
MSMFDFA of beating rate variability in isolated atrial preparations and isolated SAN myocytes. **(A–C)** heat maps for MSMFDFA scale exponents $\left(\alpha_{q}\left(s\right)\right)$ as a function of order (q) and scale for −5<q<5 and 3s<scale<415s in baseline low activity *in vivo* (*n* = 7) **(A)**, in isolated atrial preparations (*n* = 7) **(B)**, and in isolated SAN myocytes (*n* = 6) **(C)**. **(D–F)** magnification of the regions marked with white rectangles in panels A–C for the HRV signal in baseline low activity *in vivo* **(D)** in isolated atrial preparations **(E)**, and in isolated SAN myocytes **(F)** for 3s<scale<80s . **(G, H)** Statistical comparison of the MSMFDFA scale exponent of the HRV and beating rate variability signals in baseline low activity *in vivo* vs. isolated atrial preparations **(G)**, and isolated SAN myocytes **(H)** by Student’s *t*-test for −5<q<5 and 3s<scale<415s . **(I)** Statistical comparison of the MSMFDFA scale exponent of beating rate signals in isolated atrial preparations vs. isolated SAN myocytes by Student’s *t*-test for −5<q<5 and 3s<scale<415s . **(J, K)** Statistical comparison of the MSMFDFA scale exponent of HRV and beating rate variability signals in baseline low activity *in vivo* vs. isolated atrial preparations **(J)**, and isolated SAN myocytes **(K)** by Student’s *t*-test for −5<q<5 and 3s<scale<80s . **(L)** Statistical comparison of the MSMFDFA scale exponent of beating rate variability signals in isolated atrial preparation vs. isolated SAN myocytes by Student’s *t*-test for −5<q<5 and 3s<scale<80s .

**FIGURE 6:**
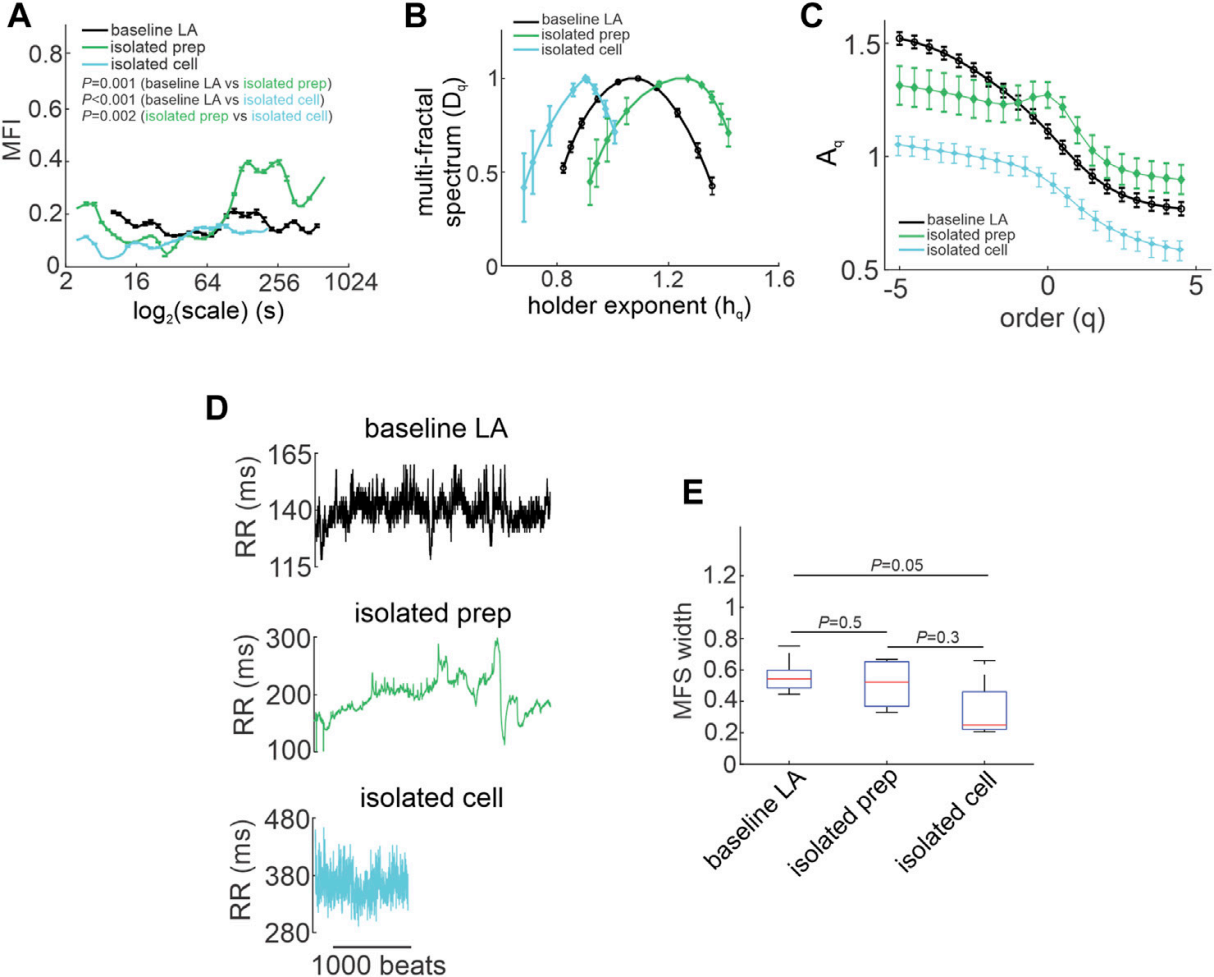
Quantification of multi-fractality in isolated atrial preparations and isolated SAN myocytes. **(A)** Multi-fractality index over 3s<scale<415s and qr=5 for baseline low activity *in vivo*, isolated atrial preparations and isolated SAN myocytes. Data analyzed by two-way repeated measures ANOVA. **(B)** Multi-fractal spectrum of the HRV and beating rate variability signals in baseline low activity *in vivo*, isolated atrial preparations and isolated SAN myocytes. **(C)** Leveling of the q-order multi-fractal exponent for negative *q* values in the beating rate variability signal of isolated atrial preparations (*n* = 7) and isolated SAN myocytes (*n* = 6). **(D)** Representative tachograms for baseline low activity *in vivo*, isolated atrial preparations and isolated SAN myocytes. **(E)** Multi-fractal spectrum width of the HRV and beating rate variability signals in baseline low activity *in vivo*, isolated atrial preparations, and isolated SAN myocytes. Data analyzed by one-way ANOVA with a Holm-Sidak posthoc test.

To directly investigate the role of the SAN in HRV fractality, MSMFDFA was performed on beating rate variability signals in isolated mouse atrial preparations (devoid of ANS inputs) and in isolated mouse SAN myocytes (Figures 5, 6). For scales>80 ms, $\alpha_{q}\left(s\right)$ was larger in isolated atrial preparations compared to baseline low activity condition *in vivo* (Figures 5A,B). MSMFDFA scale exponents were smaller in isolated SAN myocytes compared to isolated atrial preparations (Figures 5B,C). The short-range low amplitude strong fractals evident on the MSMFDFA heat map for baseline low activity (*in vivo*) at $\alpha_{q}\left(s\right)\geq 1.5$ , scales<15 s and q < 0 (Figure 5D) are not present in the signals recorded in isolated atrial preparations or isolated SAN myocytes (Figures 5E,F). Furthermore, for 18 s < scale<80 s, $\alpha_{q}\left(s\right)$ is greater in isolated atrial preparations and smaller in isolated SAN myocytes compared to baseline low activity conditions *in vivo* (Figures 5E,F). Finally, at scale<80 s, the MSMFDFA scale exponents of isolated SAN myocytes are smaller compared to isolated atrial preparations (Figures 5E,F). The statistical analysis for each of these comparisons are illustrated in Figures 5G–L.

The multi fractal index was smaller in isolated atrial preparations (at scale<64 s) and in isolated SAN myocytes (at scale<250 s) compared to baseline low activity conditions *in vivo* (Figure 6A). This indicates less variability in scale exponents of the beating rate variability in isolated atrial preparations and SAN myocytes compared to those for HRV *in vivo* when ANS signaling is intact.

Consistent with the effects of combined injection of atropine and propranolol *in vivo* (Figure 4H) the multi-fractal spectrum of the beating rate variability signal in isolated atrial preparations and isolated SAN myocytes were each asymmetric with right tail truncations (Figure 6B). Isolated atrial preparations and SAN myocytes each displayed a leveling of the q-order multi-fractal exponent for negative *q* values (Figure 6C) indicating that in the absence of ANS signaling the q-order DFA is insensitive to the magnitude of local fluctuations with small magnitudes. Representative tachograms demonstrate larger variability in beat-to-beat intervals in isolated atrial preparations and isolated SAN myocytes compared to baseline low activity conditions *in vivo* (Figure 6D). Finally, the width of the multi-fractal spectrum was shorter in isolated SAN myocytes compared to baseline low activity *in vivo* and similar between isolated atrial preparations and baseline low activity conditions *in vivo* (Figure 6E).

## Discussion

The goal of the present study was to assess the nonlinear fractality of HRV *in vivo* and in isolated preparations (intact atrial preparations and SAN myocytes) in adult healthy mice. Furthermore, we sought to assess the contributions of changes in ANS activity as well as intrinsic SAN function to the fractal structure of the HRV. Initially, we used time and frequency domain analysis to demonstrate changes in HRV during ANS blockade *in vivo* as well as in isolated atrial preparations and isolated SAN myocytes, that are typical of those shown in previous studies (Moghtadaei et al., 2017; Dorey et al., 2020; Dorey et al., 2021; Dorey et al., 2022). However, nonlinear HRV analysis using MSMFDFA provided additional insight not possible with traditional approaches. This is because traditional time and frequency domain analyses give static values for HRV while MSMFDFA generates a wide spectrum of fractal structures that provide more comprehensive insights. Specifically, while traditional HRV measures can determine if HRV is reduced, MSMFDFA can determine the structure of the variability as well as fractal characteristics. MSMFDFA is also well suited to detecting more subtle differences between experimental conditions. For example, the differences between time and frequency domain HRV measures during high and low activity phases are relatively small; however, they clearly have different fractal spectra as shown in Figure 1.

Our results demonstrate that the fractal properties of the HRV signal vary from point to point along the ECG time series, leading to multi-fractality. We quantified the MSMFDFA scale exponents on a wide range of scale (3 − 415 s) and fractal order (-5<q < 5) and our results describe the dependance of the fractal properties with different orders on the time scale in which the multi-fractality is measured. This time scale is related to the ultra-low and very-low frequency band of the HRV signal, portions of which are not quantifiable using traditional frequency domain analysis.

Our study demonstrates that PNS blockade and complete ANS blockade *in vivo* each create relatively stable and less variable fractal structures in HRV resulting in weaker multi-fractality when compared to baseline (low activity) conditions *in vivo*. Similar observations were made in isolated atrial preparations and isolated SAN myocytes, confirming that these patterns are associated with the loss of ANS (particularly PNS) signaling to the heart. In healthy mice, in baseline low activity conditions, the multi-fractal spectrum of the HRV signal was symmetrical with relatively large variability in MSMFDFA scale exponents ranging from $\alpha_{q}\left(s\right)=0.7$ to $\alpha_{q}\left(s\right)=1.6$ (as demonstrated in heat maps and by quantification of MSF width) indicating that both random behavior as well as a combination of weak and strong fractals exist in the HRV signal at baseline. Conversely, the multi-fractal spectrum of the HRV signal following ANS blockade was asymmetric with low variability of MSMFDFA scale exponent values and reduced MSF width. In the case of combined ANS blockade, MSMFDFA scale exponents ranged from $\alpha_{q}\left(s\right)=1$ to $\alpha_{q}\left(s\right)=1.6$ , which indicates the existence of fractals of various strengths only without randomness in the HRV signal. Similarly, the multi-fractal spectrum of the beating rate variability signal in isolated SAN myocytes was also asymmetric with low variability of MSFMDFA scale exponent values and reduced MSF width. However, in this case, MSMFDFA scale exponents mainly covered the range of values that are responsible for randomness and weak fractals (i.e. from $\alpha_{q}\left(s\right)=0.7$ to $\alpha_{q}\left(s\right)=1.3$ ). Collectively, these data demonstrate the importance of variability in MSMFDFA scale exponents, in addition to HRV itself, as an indicator of a healthy, adaptive cardiovascular system.

Our study shows that when the ANS is blocked or absent, the short-range, low amplitude fractals that were present and strong *in vivo* in baseline low activity conditions are eliminated. This was evident in high activity conditions *in vivo* and after ANS blockade as well as in isolated atrial preparations and isolated SAN myocytes. This indicates that the presence of short range, low amplitude fractals is a marker of intact ANS signaling with normal sympatho-vagal balance.

We found that blocking PNS signaling with atropine induces high amplitude short range fractals (scales<15 s). This is consistent with previously reported studies in healthy humans showing increases in traditional DFA $\alpha_{1}$ after atropine or glycopyrrolate administration (Tulppo et al., 2001; Perkiomaki et al., 2002; Perkiomaki et al., 2005; Castiglioni et al., 2011). Atropine also induced low amplitude fractals in the range of 15–50 s, but eliminated high amplitude, long range (scale>300 s), low amplitude, mid-range (100 s < scale<200 s), and short-range fractals (scale<10 s).

Additionally, our data show that multi-fractal HRV is primarily associated with PNS modulation and less with SNS modulation. This suggests that, in addition to its well-known role in regulating HRV at high frequency time scales (Billman, 2011), the PNS also critically regulates the ultra-low and very-low frequency components of HRV. These findings are consistent with previous studies in healthy humans (Nunes Amaral et al., 2001; Struzik et al., 2004; Nakamura et al., 2016; Shaffer and Ginsberg, 2017). In agreement with this, we found that SNS blockade with propranolol did not significantly change the MSMFDFA spectrum, multi fractal index, and multi fractal spectrum of the HRV signal. The only substantial effect of propranolol was to eliminate low amplitude short-range fractals, which is consistent with previous studies in patients (Yamamoto and Hughson, 1994; Castiglioni et al., 2011).

Following combined injection of atropine and propranolol, we found that the HRV signal became fractal on all scales and orders investigated. The only exception was the short-range, low amplitude fractals that were eliminated after combined atropine and propranolol injection. As the MSMFDFA spectrum becomes more uniform with less variability, having fractals over the entire parameter space, multi-fractality strength was clearly reduced during ANS blockade. Consistent with this, previous studies demonstrate that fractal-like behavior remains in dogs with complete denervation (Li et al., 2008).

Consistent with the effects of ANS blockade *in vivo*, the present study demonstrates that MSMFDFA scale exponents were larger in isolated atrial preparations compared to baseline low activity conditions *in vivo*. In agreement with this, the statistical comparison of the isolated atrial preparation to baseline low activity *in vivo* illustrates differences in similar regions to those observed when comparing ANS blockade *in vivo* with baseline low activity *in vivo*. This is consistent with the observation of substantially steeper slopes in denervated hearts in previous studies (Bigger et al., 1996; Perkiomaki et al., 2000). In addition, the existence of self-similar scale-free correlation (i.e. fractals in action potential firing intervals in SAN myocytes regardless of ANS receptor stimulation) have been previously reported (Yang et al., 2021). Using MSMFDFA we also demonstrate fractals with a wide range of characteristics (i.e. strengths and amplitudes) in isolated SAN myocytes, isolated atrial preparations, and in intact hearts during ANS blockade. These data support the conclusion that fractal behavior is generated, at least in part, from the intrinsic properties of the SAN. However, in isolated SAN myocytes, the MSMFDFA scale exponents were smaller compared to both isolated atrial preparations and baseline low activity conditions *in vivo*. This is indicative of the importance of both ANS signaling and the intrinsic interactions between cells in the intact SAN in determining the power law slope. The smaller multi fractal index in the beating rates of isolated atrial preparations and isolated SAN myocytes (at scale<250 s) compared to heart rate at baseline *in vivo* indicates less variability in scale exponents in the absence of ANS activity. These findings are consistent with the effects of ANS blockade *in vivo*.

The asymmetric multi-fractal spectrum of the HRV signal with right truncation in isolated atrial preparations, isolated SAN myocytes and *in vivo* following ANS blockade demonstrates that the absence of ANS signaling causes a reduction in local HRV fluctuations with small magnitudes. On the other hand, the asymmetric multi-fractal spectrum of HRV after atropine injection with left truncation suggests blocking PNS signaling alone reduces high amplitude fluctuations in the HRV signal.

Based on our data, variability in the beating rate of SAN myocytes was random (i.e. $\alpha_{q}\left(s\right)<1$ for the majority of parameter space as demonstrated in the related heat maps); however, when the level of organization was increased in a network of interconnected myocytes (i.e. in an isolated atrial preparation) variability became more structured and followed the fractal order (i.e. became non-random), which was confirmed by having $\alpha_{q}\left(s\right)>1$ for the majority of parameter space in the heat maps. While strong fractals were formed at this level of organization the multi-fractality remained low. At the next level of organization (i.e. the intact mouse) the ANS regulated this high fractality and made it more robust over a wide range of fractals while increasing multi-fractality strength. In the intact SAN, local intracellular Ca^2+^ releases from the sarcoplasmic reticulum that occur within SAN myocytes differ in spatial distribution, frequency, amplitude and phase, which results in a complex pattern of pacemaker cell excitation (Bychkov et al., 2020). Consistent with this, our data indicate that variability in beating rate of SAN myocytes is random; however, as a network of cells comes together as an ensemble in the atrial preparation, the complex interaction between cells leads to more structured variability that follows the fractal order rather than randomness. Innervation of the heart by the ANS *in vivo* regulates this high fractality and leads to a robust state with a wide range of fractal orders and randomness.

Some limitations of our study should be noted. Standard laboratory temperatures (∼22°C) could impose a cold stress on mice that may impact HR and HRV (Axsom et al., 2020). Future studies could investigate the impacts of temperature on MSMFDFA and HRV. In addition, SAN function can be affected by numerous neurotransmitters and peptides (Macdonald et al., 2020), some of which could impact HR and HR as assessed using MSMFDFA. Furthermore, the intracardiac nervous system and neurotransmitters produced within epicardial cardiac ganglia (Armour, 2007) could each have impacts on HR and HRV. These will be important areas for future study.

In summary, we have combined the use of multi-scale and multi-fractal detrended fluctuation analysis to assess the nonlinear dynamics of HRV in healthy mice. By conducting studies *in vivo* (with and without ANS blockade), in isolated atrial preparations, and in isolated SAN myocytes we were able to assess the contribution of the ANS and intrinsic SAN function to the complex, non-linear HRV dynamics in the ultra-low and very-low frequency bands. Our data demonstrate that the ANS and intrinsic SAN function each contribute to the multi-fractality of the HRV signal in healthy mice with increasing levels of structured variability as the level of organization increased from isolated cells to intact mice. Applying these approaches to mice *in vivo* and in isolated preparations enabled important insight into the regulation of complex, non-linear HR dynamics. Our data demonstrate the importance of variability in MSMFDFA scale exponents (in addition to HRV itself) for the maintenance of a healthy cardiovascular system. These analyses and approaches advance our understanding of the basis for HRV in the very-low and ultra-low frequency ranges in healthy mice and establishes an effective approach for assessing these properties across multiple scales, which is essential for a complex signal such as HR. As HR regulation shows trans-species self-similarity (Tagirova Sirenko et al., 2021) the findings and approaches identified in the present study could be applied and compared with future studies in humans. Future studies will also be able to apply MSMFDFA analysis to mouse models of disease or aging in order to better understand the roles of the ANS and intrinsic SAN function in nonlinear HRV dynamics in the ultra-low and very-low frequency bands in these conditions.

## Data Availability

The original contributions presented in the study are included in the article/supplementary material, further inquiries can be directed to the corresponding author.
